# Monitoring the performance of the Expanded Program on Immunization: the case of Burkina Faso

**DOI:** 10.1186/1472-698X-9-S1-S12

**Published:** 2009-10-14

**Authors:** Abel Bicaba, Slim Haddad, Moussa Kabore, Emile Taminy, Marta Feletto, Pierre Fournier

**Affiliations:** 1 Société d'Études et de Recherche en Santé Publique (SERSAP), Ouagadougou, Burkina Faso; 2 Fondation pour le Développement Communautaire, Ouagadougou, Burkina Faso; 3 École Nationale de Santé Publique du Centre Ouest (Koudougou), Burkina Faso; 4 Centre de Recherche du Centre Hospitalier de l'Université de Montréal, Montreal, Canada

## Abstract

**Background:**

The greatest challenge facing expanded programs on immunization in general, and in Burkina Faso in particular, lies in their capacity to achieve and sustain levels of immunization coverage that will ensure effective protection of children. This article aims to demonstrate that full immunization coverage of children, which is the primary indicator for monitoring national immunization programs, is sufficient neither to evaluate their performance adequately, nor to help identify the broad strategies that must be implemented to improve their performance. Other dimensions of performance, notably adherence to the vaccination schedule and the efficacy of the approaches used to reach all the children (targeting) must also be considered.

**Methods:**

The study was carried out using data from surveys carried out in Burkina Faso: the 1993, 1998 and 2003 Demographic and Health Surveys and the 2003 national Survey of Immunization Coverage. Essentially, we described levels of immunization coverage and their trends according to the indicators considered. Performance differences are illustrated by amplitudes and maximum/minimum ratios.

**Results:**

The health regions' performances vary according to whether they are evaluated on the basis of full immunization coverage or vaccination status of children who have not completed their vaccinations. The health regions encompass a variety of realities, and efforts of substantially different intensity would be required to reach all the target populations.

**Conclusion:**

Decision-making can be improved by integrating a tripartite view of performance that includes full immunization coverage, adherence to the vaccination schedule (timely coverage), and the status of children who are not fully vaccinated. With such an approach, interventions can be better targeted. It provides information on the quality and timeliness of vaccination and identifies the efforts required to meet the objectives of full immunization coverage.

**Abstract in French:**

See the full article online for a translation of this abstract in French.

## Abstract in French

See Additional file 1 for a translation of the abstract to this article in French.

## Background

Substantial efforts have been made by sub-Saharan African countries to reinforce their immunization programs. One of the major challenges facing immunization services is to achieve and sustain the high levels of performance necessary for complete and appropriate coverage of target populations [1-3]. Usually, the monitoring of immunization services performance is done by compiling and analyzing indicators of completeness. These show the extent to which immunization programs were able to vaccinate targeted children against the entire group of antigens targeted by the Expanded Program on Immunization (EPI): BCG, diphtheria, tetanus, pertussis, poliomyelitis, measles, and yellow fever. The measures generally used indicate the proportion of children who are fully vaccinated (full immunization coverage (FIC)) or vaccinated for specific antigens (particularly DTCP3) [4,5]. This process is restrictive, however, in that it only takes into account the number of vaccines received, but not the age of the child at the time of vaccination and the adherence to the vaccination schedule. The use of FIC as an indicator of the effectiveness of immunization programs leads to categorizing targeted children into two groups: those fully vaccinated and those not fully vaccinated. This dichotomy has the advantage of simplicity, but it oversimplifies the reality, which is that both categories include a broad spectrum of different situations in terms of vaccination efficacy and adequacy. It results, for example, in children who are fully vaccinated being considered "successes" whether or not the vaccination schedule was adequately respected. This group thus includes children vaccinated too late or too early, whose chances of being really immunized are thereby limited. When all is said and done, the homogeneity of the "fully vaccinated" group of children is only ostensible, and health regions that are considered to be high-performing in terms of coverage may actually be performing at a mediocre level if we take into account the children's age at vaccination and the degree to which the vaccination schedules of the target populations were respected. This is confirmed by several studies carried out in sub-Saharan Africa. In Malawi, a recent study shows that while 93% of children had received their third dose of DTC by the age of 23 months, only 2% had received it at the recommended age of 14 weeks, and 49% at six months [6]. The same trend is seen with both BCG (95% coverage at 15 months, only 22% at one week and 53% at six weeks) and measles. In the Central African Republic, Kahn et al. reported a pattern of late vaccination for measles [7]. They calculated that vaccinated children who had an opportunity for earlier vaccination were, on average, exposed to 70 "days-at-risk of measles" (age at vaccination in days minus 270, which is the recommended age for vaccination). In rural areas, the average delay was more than three months (98 days). Other studies mention vaccinations that are too early. A survey in Mozambique reports that among all children who had received all required vaccines, one out of 10 had received the measles vaccine before 8.5 months of age [8]. In the previously mentioned study in Malawi, 17% were vaccinated against measles before nine months of age.

In addition, this dichotomy does not take into account possible disparities in vaccination status among the group of children considered not fully vaccinated. It obscures the fact that some children in this group have received no vaccine at all, while others are missing only one or two, to have full coverage. Thus, regions with good FIC performance can show poor performance in their capacity to improve this indicator, since the proportions are high of children with no vaccine and those missing one or two. Therefore, analyzing the outcomes of immunization activities and evaluating program performance require using an array of indicators that take into consideration both quantitative (ability to reach targeted children) and qualitative (respecting the vaccination schedule for children reached in the targeted population) dimensions of vaccination.

In this article, we propose an approach to performance analysis based on the efficacy of the targeting of children to be vaccinated. Appropriate targeting is defined by the EPI's capacity, on the one hand, to reach the targeted children, and on the other, to vaccinate those children in accordance with the vaccination schedule. We intend to show that such an approach, simple in its application and requiring no particular skills of analysis, makes it possible to go beyond the general indications provided by the rough indicators of coverage and obtain a truer picture of the real immunization activities in regions or health districts. It offers the potential to uncover territorial entities whose actual poor performance is masked by levels of gross immunization coverage that appear satisfactory, and, in so doing, contributes more effectively to the development of strategic orientations. However, in this paper, which is essentially focused on the development and utilization of more precise performance indicators for vaccination programs, we will not deal with the reasons underlying why vaccination schedules are not respected and coverage is adequate or inadequate. The reader interested in these issues may consult the scant literature on this topic, particularly the studies of Jahn in Malawi [6].

## Methods

### Sources of data

The data we used were taken from three Demographic and Health Surveys (DHS) -- 1993, 1998, 2003 -- and the Immunization Coverage Survey (ICS) carried out in 2003 at the level of health districts in Burkina Faso. The DHS is a national survey carried out using a standardized methodology [9-11]. The surveys of 1993 and 1998 used a division of the country into five economic regions while that of 2003 was based on the country being divided into 13 administrative regions that correspond also to the health regions. The samples of the three surveys are briefly described in Table 1.

**Table 1:** Demographic and Health Surveys (DHS) in Burkina Faso: Sample and territorial representation.

Year	Territorial representation	Sample (*n*)
1993	5 economic regions	1104
1998	5 economic regions	1041
2003	13 health regions	1840

The ICS was based on the cluster method developed by WHO [12,13]. Each cluster consists of at least seven children between the ages of 12 and 23 months and seven mothers of babies from 0 to 11 months of age. The sampling frame was the population of each health district: 30 clusters were selected per health district, and thus, at the national level, there were 1560 clusters. Sampling took into account the demographic importance of the villages and the sectors of communes, thereby ensuring good proportional representation. The identification of clusters was done using COSAS software and the National Health Information System file of the Direction des études et de la Planification. In each cluster selected, the seven children are identified in households by moving from one relation to another, extending outward from a central point.

### Vaccination schedule, evaluation criteria

The vaccination schedule used in Burkina Faso during the period covered by the three DHSs and the ICS is presented in Table 2. The immunization coverage rates that are considered in the DHSs and the ICS are estimated from the children's health booklets presented by the mothers or, for the BCG vaccine, from the surveyor's direct observation of a scar. A child's coverage is considered to be complete if he has received one dose of BCG, three of DTP, one of measles vaccine, and one of yellow fever vaccine. The child is considered to have been correctly vaccinated (timely vaccination) if the minimum interval between two doses of DTP is 28 days and if the child was vaccinated for measles and yellow fever no sooner than 255 days after birth.

**Table 2:** Burkina Faso: Vaccination schedule.

Contacts	Age at vaccination	Recommended antigens
1	Birth	BCG, polio 0
2	2 months	DTCP1, polio 1
3	3 months	DTCP2, polio 2
4	4 months	DTCP3, polio 3
5	9 months	Measles, yellow-fever vaccines

### Analysis

Full immunization coverage is the primary indicator used to illustrate EPI performance (at the national and regional levels) and its evolution over the three periods (1993, 1998, and 2003). The second indicator is timely coverage. We used two scenarios for timely coverage in order to better assess the requirements entailed by adherence to the vaccination schedule. For a child to be considered correctly vaccinated, fixed time intervals were set for administration of the DTCP and the measles vaccines, as well as the yellow fever vaccine. Thus, in the first scenario we consider the vaccination to be timely if the interval between the first and third doses of DTCP is less than 60 days, and if the vaccine for measles and yellow fever is administered no sooner than 255 days and no later than 360 days after birth. The second scenario is less conservative: we consider vaccination to be timely if the interval between the first and third doses of DTCP is less than or equal to 90 days, with the same requirements as above for the measles and yellow fever vaccines. Comparative analysis of the evolution of the FIC and of timely coverage is carried out at the regional level for 1993 and 1998. Finally, to compare the performances of the health regions, we use a third group of three indicators that describe the vaccination status of children who have not completed their vaccinations: percentages of children who have received no vaccine, have missed only one, or have missed two vaccinations on the schedule.

## Results

The estimate of FIC for 2003 provided by the ICS is 52.2%, with quite substantial regional variations. The estimates provided by the DHSs are more conservative and reveal a positive progression of immunization coverage in the period 1998-2003 (Table 3). All the rough indicators of coverage converge and indicate an increase in coverage for the various antigens, a decrease in children lost to follow-up of BCG/DTCP1 and DTCP1/DTCP2, and a relative reduction in regional disparities. However, the picture presented by the rough indicators of coverage is much less impressive once the quality of the targeting is taken into account. Even the least conservative scenario reveals a disturbing situation. With barely one child out of five having been correctly and appropriately vaccinated in 2003 and, among those fully vaccinated, scarcely one out of two having been vaccinated in accordance with the norms of the vaccination schedule, population coverage and national EPI performance remain limited.

**Table 3:** Vaccination coverage in Burkina Faso 1993-2003.

	Source of information			
	
Indicator	ICS2003	DHS2003	DHS1998	DHS1993
National immunization coverage				
FIC (full immunization coverage)	52.1	44.0	28.8	34.0
BCG	90.3	81.0	72.6	86.3
DTCP3	77.0	57.0	41.3	40.6
Measles	71.6	56.0	49.0	61.1
Regional disparities in immunization coverage (FIC)				
Region with lowest FIC	31.0	33.1	7.1	-
Region with highest FIC	72.0	61.6	37.7	-
Ratio Max/Min	2.3	1.9	5.3	-
Rate of loss to follow-up				
DTCP1/DTCP3	-	18.3	31.6	48.3
BCG/DTCP3	-	21.2	39.1	52.3
Appropriateness of targeting				
DTCP1: children vaccinated before their 3rd month of life	-	47.9	37.6	28.2
DTCP3: children vaccinated before their 5th month of life	-	27.5	22.5	18.8
Measles: children vaccinated between the 9th and 12th months of life	-	73.5	67.8	67.4
Children correctly vaccinated among those completely vaccinated - Scenario 1	-	36.2	27.8	26.6
Children correctly vaccinated among those completely vaccinated - Scenario 2	-	55.5	46.8	45.6
Children completely and correctly vaccinated - Scenario 1	-	14.1	7.3	7.9
Children completely and correctly vaccinated - Scenario 2	-	21.5	12.2	13.6

*Children who received the first vaccination cited, but not the second.

**Figure 1:**
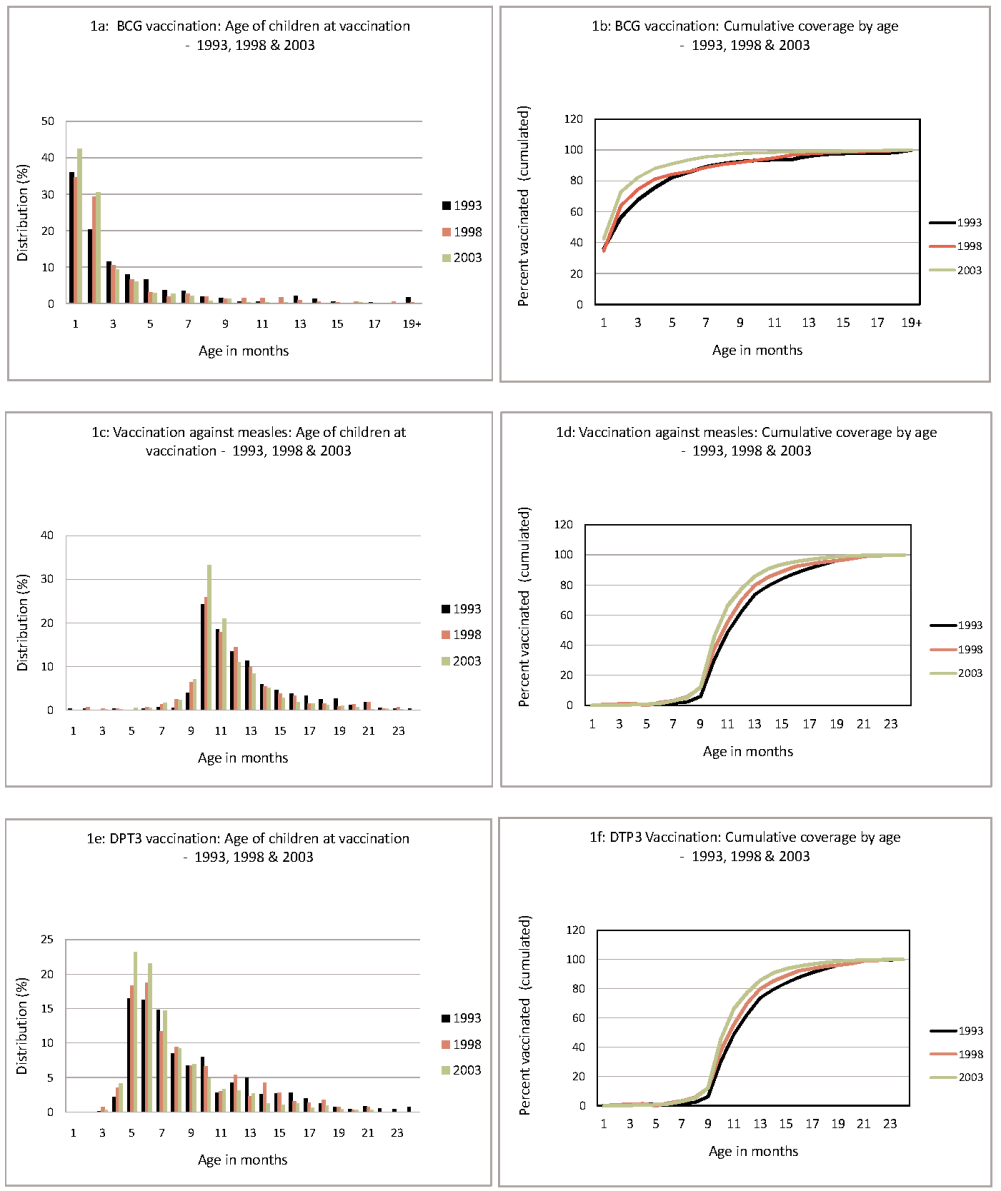
**Age of children at vaccination and cumulative coverage by age: BCG, Measles, DTCP3 - 1993, 1998 and 2003**.

The current discrepancy between complete coverage and timely coverage is largely due to the late vaccination of children, as shown in Figures 1a to 1e. Thus, in 2003, fewer than half (42.5%) of the children vaccinated with BCG received it in their first month of life (Figure 1a), and 18% had received it after the age of three months (Figure 1b). More than one-third (36%) of vaccinated children had received their third dose of DTCP after the age of six months, and 20% after the age of nine months (Figures 1e, f). Finally, only 7% of children vaccinated against measles were vaccinated at the age of nine months; 5% were vaccinated before nine months, 65% between months 10 and 12, and 17.4% at 13 months and over (Figures 1c, d). However, the six figures show -- and it is here that we see the value of the indicators used -- that the quality of targeting of children improved significantly between 1998 and 2003 for each of the three vaccinations (Table 4). In the case of BCG, for example, children are vaccinated earlier and earlier, and the vaccination schedule is better respected. The low rates of coverage in assisted deliveries in health facilities partly explain why half the children receive their BCG vaccine late, at the age of one month. The situation is also improving for measles and DTCP3.

**Table 4:** Age (months) at vaccination of children for BCG, measles and DTCP.

Year	1993	1998	2003
BCG			
Total vaccination coverage (%)	70.0	49.4	59.5
Mean age at vaccination	2.6	2.3	1.5
Median age at vaccination	1.0	1.0	1.0
Standard deviation	3.9	3.4	2.4
Measles			
Vaccination coverage	50.9	35.3	45.4
Mean age at vaccination	11.4	10.8	10.3
Median age at vaccination	11.0	10.0	10.0
Standard deviation	3.2	3.1	2.5
DTCP1			
Vaccination coverage	66.8	45.7	59.8
Mean age at vaccination	4.8	4.5	3.5
Median age at vaccination	3.0	3.0	3.0
Standard deviation	3.8	3.8	2.9
DTCP2			
Vaccination coverage	55.8	39.1	55.3
Mean age at vaccination	7.1	6.2	5.2
Median age at vaccination	6.0	5.0	4.0
Standard deviation	4.0	3.8	3.1
DTCP3			
Vaccination coverage	34.4	31.7	48.9
Mean age at vaccination	8.0	7.5	6.6
Median age at vaccination	7.0	6.0	6.0
Standard deviation	4.2	3.8	3.2

Source: DHS 1993, 1998, 2003.

Table 5 presents the performance, in terms of immunization coverage, of the country's five large territories in 1993 and 1998 (comparable data are unfortunately not available for 2003 because of the administrative reconfiguration of the regions). Whatever indicator is used, the performances of the regions vary considerably. The central region, which includes the urban region of the capital, presents levels of performance significantly higher than the others (Table 5). In every case, the proportion of children who are correctly vaccinated appears low, regardless of the region, although there has been a tendency toward improvement over the years. The performances of the regions are better if we consider the longer intervals between the first and third doses of DTCP, which reflect the tendency to vaccinate somewhat later those children who have already received their first dose of DTCP. The administrative reports we consulted suggest that this will be repeated in 2003 [12], but always with the considerable disparity between the central region and the rest of the country.

**Table 5:** Vaccination coverage by large regional groupings in 1993 and 1998.

	1993			1998		
		
		Timely coverage			Timely coverage	
				
Region	Gross coverage (FIC)	Scenario 1	Scenario 2	Gross coverage (FIC)	Scenario 1	Scenario 2
Center	60.4	29.4	43.5	69.1	38.2	51.8
North	11.4	2.2	2.7	20.3	4.1	10.1
East	32.1	5.7	13.4	23.1	7.2	9.5
West	32.3	6.0	11.4	19.2	2.9	4.5
Center-South	27.9	8.1	13.4	33.1	7.0	15.2

Scenario 1: Children completely and correctly vaccinated (measles at 9-12 months and DTCP 1-3 intervals of ≤ 2 months).

Scenario 2: Children completely and correctly vaccinated (measles at 9-12 months and DTCP 1-3 intervals of ≤ 3 months).

Source: DHS 1993, 1998.

**Figure 2:**
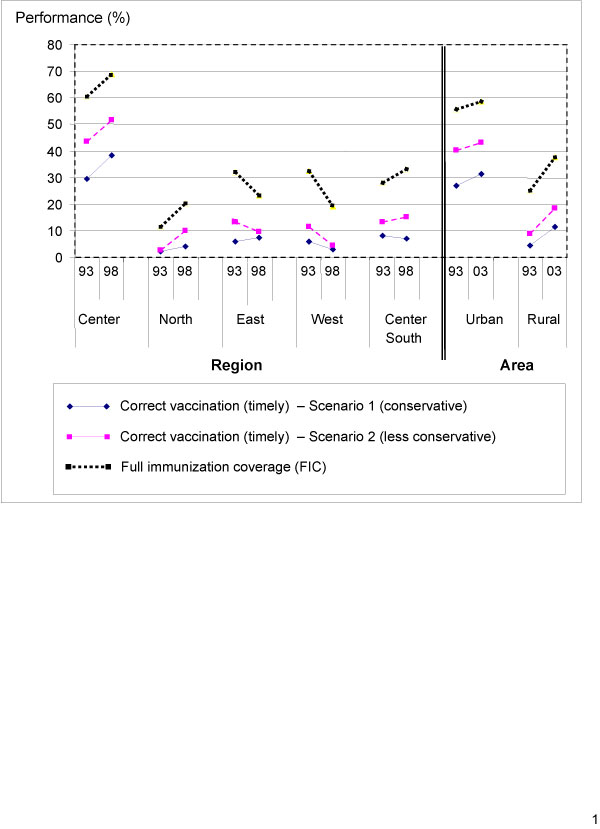
**Evolution of FIC and appropriate coverage by regional groupings**.

It is interesting to note that the regions where FIC is greatest are also those where the vaccination schedule is generally better respected, and inversely (Figure 2), in both urban and rural areas. One exception to this is in the East region. As with FIC, the proportion of children correctly vaccinated in the rural area is considerably lower than in the urban area. In both areas, there was significant improvement between 1993 and 2003. The fact that both FIC and timely coverage are moving in the same direction is an encouraging sign (Figure 2).

Table 6 presents regional performance with respect to the vaccination status of children who had not yet completed their vaccinations in 2003 (source: ICS). A first group consists of three health regions where a high proportion (more than 40%) of children with incomplete vaccination actually had no vaccinations at all. These are the regions of Hauts-Bassins, Plateau-Central, and Sahel. In other words, in these regions there are still enormous needs among the population of children whose vaccinations are incomplete. A second group is made up of regions in which a high proportion of children with incomplete vaccinations have missed only one. While coverage of these children is incomplete, most of them have completed two of the three required vaccination sequences and would need only one complementary vaccine to be completely covered. These regions are the North, Center-West, Center, and Boucle du Mouhoun. The six other regions are in an intermediate position. The children who are not completely vaccinated are distributed relatively evenly among those having received no vaccines, those missing one, and those missing two. These three groupings convey not only different levels of performance among the health regions, but also situations that will require efforts of varying levels of intensity to achieve the goals of complete coverage and to address the unmet needs of target populations effectively.

**Table 6:** Vaccination status of children with incomplete coverage by health region.

	Children not completely covered		Distribution of children whose vaccinations are incomplete (%)		
		
Region	N	%	No vaccination	Missing one vaccination	Missing two vaccinations
Hauts-Bassins	159	62	47	25	28
Plateau Central	56	55	46	31	23
Sahel	108	77	42	35	23
East	73	56	37	30	33
Cascades	32	48	35	24	41
Center-East	88	56	30	27	43
Center-North	77	41	29	30	41
Center-South	44	48	26	34	40
South-West	41	47	25	24	51
North	148	72	23	44	33
Center-West	75	64	22	30	48
Center	50	37	13	60	27
Boucle du Mouhoun	80	50	11	48	41
TOTAL	1031	100	31	34	35

Source: DHS 2003.

The regions' performances are different depending on whether we are looking at the level of FIC or the vaccination status of children who have not completed their vaccinations. Some regions perform well in terms of the former, but not the latter (notably Center-East and Center-South). Conversely, some regions have poor FIC performance, but a major part of their incomplete vaccinations consists of children who have missed only one vaccine. Thus, for example, the Boucle du Mouhoun region ranks nine out of 13 in terms of FIC, but with respect to children with incomplete vaccination, it has the lowest proportion of children who have never had a vaccine.

## Discussion

Numerous efforts have been made over recent decades to broaden the availability of vaccination services in Burkina Faso, such as a policy to achieve autonomy in vaccination by purchasing vaccines through the national budget, and extension of service coverage [12]. This article proposes a simple and practical approach to help decision makers assess EPI performance and progress, in order to identify high-priority and more specific needs for intervention.

The most commonly used indicator, FIC, allows for the evaluation, up to a certain point, of national EPI performance and its progress and of disparities among health regions. However, as we have seen, this indicator does not take into account the heterogeneity of situations encountered related to respecting the vaccination schedule, and particularly to delays in the administration of one or several vaccines. Little is known about the population impacts of these incorrect vaccinations on the outcomes of vaccination programs, which will need to be better documented. However, they may be greater than expected. In Germany, Siedler et al., found that "50% of measles cases in 1-year old children would be prevented if presently observed vaccine coverage rates in the third year of life could be achieved 12 months earlier" [14]. A delay in the administration of one vaccine could not only increase the child's vulnerability to the antigen concerned, but also weaken adherence to subsequent vaccination [15] and thereby increase the risk that the child will never complete the vaccination course [16]. Finally, a high level of incorrect vaccinations could significantly affect these programs' ability to lower vaccine-preventable mortality. In Bangladesh, Brieman et al. showed that children vaccinated with BCG before the age of six months are at considerably lower risk of dying in the first five years of life than are children vaccinated later (hazard rate: 0.47-0.73) [17].

Thus, timeliness of vaccine coverage complements the information provided by FIC. It offers a more precise rendering of EPI efficacy, provides more plausible measures of the actual immunization of target populations and thereby offers better information on the quality of processes. Where targeting is perfect, these two indicators coincide. However, this is far from the case in Burkina Faso, where it seems the priority issues are related as much to improving vaccination processes as to extending coverage. It is nevertheless interesting to note that there is a fair amount of concordance between the level of gross coverage and the quality of processes as indicated by the measurement of timely coverage (Table 5). With only one exception, this concordance is also observed in the progress of performances among the health regions at the disaggregate level.

Examining the vaccination status of children who are not fully vaccinated allows us to refine the EPI performance analysis and to determine more precisely both the scope of the needs to be addressed and the interventions required to achieve complete and timely coverage. At the national level, 31% of children who are not fully vaccinated have received no vaccine at all, which means that nearly one-third of non-vaccinated children have had no contact with immunization services. At the regional level, the high proportion of children having had no vaccine among those not fully vaccinated represents a problem of inadequate immunization strategies. The effort required to respond to the needs of these children is substantial. Why are so many children never reached? To what extent are these children concentrated in populations that are poor, far from points of service, or vulnerable, and also, to what extent do these children lost to follow-up reflect existing health inequities? More detailed analysis of local strategies would help in understanding the reasons and the systemic deficiencies at play. Conversely, in the regions where the proportion of children who have received no vaccine is low, the problem has less to do with the appropriateness of the overall strategy and the quality of the processes for reaching targeted children, than with the capacity of the services in place to guarantee the completeness of the vaccinations carried out.

Our results show that, in the regions, the level of complete coverage is poorly correlated with the proportion of children not vaccinated at all, and consequently, it is not very useful for estimating the scope of work to be carried out (Table 7). Comparing the ratings of the regions on the basis of the three performance criteria we used reveals not only their complementarity, but also the limitations of any analysis of coverage that is focused exclusively on complete coverage. Some regions that rate well on the FIC criterion are in a lower position when it comes to the proportion of children who received no vaccine at all. Conversely, the ratings of regions such as the North, Center-West, and Boucle du Mouhoun improve when we move from FIC to children with no vaccine. Finally, some regions such as Hauts-Bassins, Sahel, and South-West have relatively stable ratings.

**Table 7:** Ratings of health regions according to three performance criteria.

	Rating		
	
Region	Level of FIC	Proportion of children with no vaccine	Level of timely coverage
Center	1	2	2
Center-North	2	7	3
Center-South	3	6	1
Center-East	4	8	4
South-West	5	5	12
East	6	10	6
Cascades	7	9	8
Plateau Central	8	12	11
Boucle du Mouhoun	9	1	10
Hauts-Bassins	10	13	7
Center-West	11	3	9
North	12	4	5
Sahel	13	11	13

These results indicate that, in analyzing EPI performance, taking into consideration both FIC and targeting will provide better vantage points from which decisions can be taken to improve the situation. In certain regions such as Sahel, Hauts-Bassins, and Plateau-Central, not only is FIC low, but the outlook for improvement is poor because of the inadequate strategies in place. In the Center-North, Center-East, and East regions, the current inadequacy of the immunization strategies will eventually make it difficult to improve complete coverage which is, for the moment, relatively good. On the other hand, in the regions of Boucle du Mouhoun, North, and Center-West, the results suggest that the current low level of FIC could be improved quite significantly by placing greater emphasis on improving the efficacy of current strategies for completing vaccinations.

## Conclusion

The goal of the EPI is to achieve a significant reduction in vaccine-preventable diseases. From this perspective, the national objective is to ensure complete and timely immunization coverage for 80% of targeted children [12]. In recent decades, substantial progress has been made in immunization coverage. To sustain this trend, more precise information is needed on EPI performance. The results of the present study indicate that decision-making can be improved by integrating a tripartite view of performance that includes FIC, adherence to the vaccination schedule (timely coverage), and the status of children who are not completely vaccinated. Using such an approach, interventions can be better targeted and help to reduce inequities in health care and access to vaccination. In effect, combined analysis of these three dimensions at the district, regional, and national levels enables us to identify more clearly the extent to which children have received all the doses of vaccines, and the extent to which these doses respected the vaccination schedule, as well as the exact status of those who did not receive all the doses. At this time, there are studies [18-22] that have documented the various determinants of the elements of this performance, notably with respect to adherence to the vaccination schedule and full immunization coverage. More detailed studies should make it possible to better understand the reasons underlying the performance deficiencies encountered.

## List of abbreviations used

DHS: Demographic and Health Survey; EPI: Expanded Program on Immunization; FCI: Full immunization coverage; ICS: Immunization Coverage Survey.

## Competing interests

The authors declare that they have no competing interests.

## Authors' contributions

AB coordinated the study. As principal investigators, AB and SH were responsible for all scientific aspects of the work. All authors were involved in the preparation of the research project, the analyses, and the drafting of the manuscript. MK and ET were responsible for interactions with decision makers and stakeholders. MF supervised analysis of the databases and the formulation of results. PF provided scientific support during the various phases of the study. All authors provided feedback and made revisions to the manuscript.

## Supplementary Material

Additional file 1 Abstract in French. Click here for file
