# Supplementary material for: Monitoring the performance of the Expanded Program on Immunization: the case of Burkina Faso
Source: BMC Int Health Hum Rights. 2009 Oct 14;9(Suppl 1):S12. doi: 10.1186/1472-698X-9-S1-S12 (PMC3226231; doi:10.1186/1472-698X-9-S1-S12)
Supplement: Additional file 1 — Abstract in French. [file 1472-698X-9-S1-S12-S1.pdf]

Suivi de la performance du Programme Élargi de Vaccination: le cas du Burkina Faso

Abel Bicaba, Slim Haddad, Moussa Kabore, Emile Taminy, Marta Feletto, Pierre Fournier

Résumé

Problématique

Le principal défi auquel sont confrontés les Programmes Élargis de Vaccination en général, et au Burkina Faso en particulier, réside dans leur capacité d'atteindre et maintenir des niveaux de couverture vaccinale à même d'assurer une protection efficace des enfants. Ce papier vise à démontrer que l'indicateur principal de suivi des programmes nationaux de vaccination qu'est la couverture vaccinale complète des enfants n'est pas suffisant pour évaluer adéquatement leur performance, et permettre d'identifier les stratégies à mettre en œuvre pour l'améliorer. D'autres aspects notamment le respect du calendrier vaccinal et l'efficacité des stratégies à rejoindre l'ensemble des enfants (ciblage) doivent êtres considérés pour rendre compte adéquatement des réalisations des programmes.

Méthodes

L'étude a été réalisée en utilisant des données d'enquêtes réalisées au Burkina Faso: les Enquêtes Démographiques et de Santé de 1993, 1998 et 2003, et l'Enquête Nationale de Couverture Vaccinale réalisée en 2003 suivant la méthode des grappes de l'OMS. Nous décrivons les niveaux de couverture vaccinale et leur évolution en fonction des indicateurs considérés.

Résultats

La performance des régions sanitaires n'est pas la même selon qu'on la juge sur la base du niveau de couverture vaccinale complète, ou du statut vaccinal des enfants n'ayant pas complété leur vaccination. Les régions sanitaires couvrent des réalités diverses et des efforts d'intensité substantiellement différente seraient requis pour rejoindre effectivement l'ensemble des populations cibles.

Conclusions

La prise de décision gagne à intégrer une triple perspective de la performance, considérant à la fois la couverture vaccinale complète, le respect du calendrier vaccinal (la couverture adéquate), et le statut des enfants non complètement vaccinés. Une telle démarche permet de mieux cibler les interventions. Elle fournit des informations sur la qualité et l'adéquation de la vaccination et rend compte des efforts requis pour atteindre les objectifs de couverture vaccinale complète.
